# Effects of Gold Nanoparticles Functionalized with Bioactive Compounds from *Cornus mas* Fruit on Aorta Ultrastructural and Biochemical Changes in Rats on a Hyperlipid Diet—A Preliminary Study

**DOI:** 10.3390/antiox11071343

**Published:** 2022-07-08

**Authors:** Remus Moldovan, Daniela-Rodica Mitrea, Adrian Florea, Irina-Camelia Chiş, Şoimiţa Suciu, Luminiţa David, Bianca Elena Moldovan, Laura Elena Mureşan, Manuela Lenghel, Rodica Ana Ungur, Răzvan Vlad Opriş, Nicoleta Decea, Simona Valeria Clichici

**Affiliations:** 1 Department of Physiology, Iuliu Hatieganu University of Medicine and Pharmacy, 1-3 Clinicilor Street, 400006 Cluj-Napoca, Romania; moldovan.remus@umfcluj.ro (R.M.); ichis@umfcluj.ro (I.-C.C.); mihaela.suciu@umfcluj.ro (Ş.S.); nicoletadecea@gmail.com (N.D.); sclichici@umfcluj.ro (S.V.C.); 2 Department of Cell and Molecular Biology, Iuliu Hatieganu University of Medicine and Pharmacy, 6 Pasteur Street, 400349 Cluj-Napoca, Romania; aflorea@umfcluj.ro (A.F.); opris.razvan93@gmail.com (R.V.O.); 3 Research Center for Advanced Chemical Analysis, Instrumentation and Chemometrics, Faculty of Chemistry and Chemical Engineering, Babes-Bolyai University, 11 Arany Janos Street, 400028 Cluj-Napoca, Romania; luminita.david@ubbcluj.ro (L.D.); bianca.moldovan@ubbcluj.ro (B.E.M.); 4 Raluca Ripan Institute of Research in Chemistry, Babes-Bolyai University, 30 Fantanele Street, 400294 Cluj-Napoca, Romania; laura.muresan@ubbcluj.ro; 5 Radiology Department, Iuliu Hatieganu University of Medicine and Pharmacy, 1–3 Clinicilor Street, 400006 Cluj-Napoca, Romania; lenghel.manuela@gmail.com; 6 Department of Rehabilitation, Iuliu Hatieganu University of Medicine and Pharmacy, 8 Victor Babes Street, 400012 Cluj-Napoca, Romania; rodica.ungur@umfcluj.ro

**Keywords:** gold nanoparticles, *Cornus mas*, aorta, oxidative stress, antioxidants, endothelium

## Abstract

*Cornus mas* L. extract (CM) presents hypolipidemic, antioxidant and anti-inflammatory activity. Gold nanoparticles (AuNPs) are considered potent delivery systems and may be used to release pharmaceutical compounds at the level of injury. In our study, we used gold nanoparticles functionalized with bioactive compounds from *Cornus mas* L. (AuNPsCM) in an experimental model of a high-fat diet (HFD), and we assessed their effects on aorta wall but also in the serum, as compared to *Cornus mas* (CM) administration. Sprague Dawley female rats were fed for 9 months with an HFD. During the last month of the experiment, we randomly allocated the animals into three groups that received, by oral gavage: saline solution, CM solution (0.158 mg/mL polyphenols) or AuNPsCM solution (260 μg Au/kg/day), while a Control group received a standard diet and saline solution. At the end of the experiment, we performed an ultrasonography of the aorta and left ventricle and a histology and transmission electron microscopy of the aorta walls; we investigated the oxidative stress and inflammation in aorta homogenates and in serum and, in addition, the lipid profile. AuNPsCM presented better effects in comparison with the natural extract (CM) on lipid peroxidation (*p* < 0.01) and TNF-alpha (*p* < 0.001) in aorta homogenates. In serum, both CM and AuNPsCM decreased the triglycerides (*p* < 0.001) and C-reactive protein (CM, *p* < 0.01; AuNPsCM, *p* < 0.001) and increased the antioxidant protection (*p* < 0.001), in comparison with the HFD group. In intima, AuNPsCM produced ultrastructural lesions, with the disorganization of intima and subendothelial connective layer, whereas CM administration preserved the intima normal aspect, but with a thinned subendothelial connective layer. AuNPsCM oral administration presented certain antioxidant, anti-inflammatory and hypolipidemic effects in an experimental model of HFD, but with a negative impact on the ultrastructure of aorta walls, highlighted by the intima disorganization.

## 1. Introduction

Nowadays, a high-lipid diet represents a nutrition problem, with more and more people being affected by overweight and obesity. A prolonged high-fat diet (HFD) alters the physiological mechanisms and initiates numerous pathways in different body organs. Numerous studies present the connection between obesity, endothelial dysfunction and metabolic and cardiovascular diseases [1,2].

Atherosclerosis, the consequence of endothelium alteration, represents the main cause of cardiovascular diseases [3], and different factors trigger the pathological mechanisms that damage the endothelium.

Atherosclerosis (ATS) is a chronic inflammatory disease of the arterial wall, one of the most common diseases in adults with a negative impact on the quality of life, and a cause of morbidity and mortality, especially in older patients [4], and it may occur as a consequence of a prolonged high-fat diet (HFD) [5]. The main risk factor for atherosclerosis is hyperlipidemia, especially the increase in low-density lipoprotein (LDL) cholesterol molecules. Endothelial injury, nitric oxide (NO) imbalance, and endothelin levels are closely linked to the development of ATS [6,7].

As a result of endothelial injury, endothelial cells release inflammatory mediators that promote the progression of ATS [8]. Because it plays a major role in the pathogenesis of ATS, vascular endothelium dysfunction has become an important therapeutic target [9]. The search for remedies with hypolipidemic and/or anti-inflammatory properties, with the ability to inhibit the progression of ATS and therefore other cardiovascular diseases, is essential.

Nanotechnology developed various types of nanoparticles that may be used for industrial, medical, cosmetic and even dietary purposes, with the human body becoming thus exposed more and more to these products. Recent research considers nanoparticles to be potent delivery systems for synthetic drugs or for the natural compounds than must be transported to the target tissue cells [10,11], but some other studies have reported their toxicity to different body systems [12,13,14]. Additionally, the human body is constantly exposed to numerous environmental factors that can influence the tissues’ physiological processes. Among these, important influences that cannot be affected by humans are represented by the atmospheric nanoparticles that may easily enter into the body and alter the endothelium, leading to atherosclerosis development [15], atheroma that can be fragmented with fatal effects under specific environmental conditions [16]. There are factors that target the endothelium, and their influence can be modulated by diet or by medication. 

Gold nanoparticles (AuNPs), easy to be synthesized and functionalized [17], were used for medical purposes in many studies, being considered biocompatible and with a low toxicity on cells, compared to other types of nanoparticles [18]. The safe use of AuNPs has not been studied enough, and many questions about their efficacy and toxicity are still without answers.

Over time, many therapeutic compounds have been proposed to improve endothelial dysfunction and researchers have tested various herbal remedies to inhibit the progression or development of ATS or to reduce the main risk factor—dyslipidemia [19,20,21].

Herbal products have a lower price and a lower toxicity [22]. Among them, *Cornus mas* L. from the Cornaceae family, a plant found as native or cultivated in Central and Southern Europe and South Asia, has demonstrated not only its antioxidant and anti-inflammatory potential [23] but also its capacity to reduce hyperlipidemia [24], hypertrygliceridemia and hypercholesterolemia and to improve liver function [25], not only in experimental models, but also in patients [26], with all these properties making it a valid candidate for the amelioration of lipid profile, oxidative stress and vascular inflammation.

The main active compounds of *Cornus mas* (found in fruits, flowers or leaves) are anthocyanins, flavonoids, phenolic acids, tannins, carotenoids, vitamins and fatty acids, which present many pharmacological activities (antidiabetic, hepatoprotective, cardioprotective, anti-inflammatory, cytotoxic, etc.) [27].

Nevertheless, plant therapy has some limitations including poor lipid solubility, poor stability and reduced quantities of active compounds that reach the target site, with a low therapeutic efficacy [28,29]. Over recent years, to overpass these limitations, new systems of drug delivery have been proposed.

The prooxidant-antioxidant balance can be altered by HFDs, a process that we considered important for research investigation. Based on our previous studies with the *Cornus mas* L. but also with the gold nanoparticles functionalized with *Cornus mas* (AuNPsCM), we investigated the oxidative stress and the pathogenic mechanism triggered by hyperlipidemia. We tested previously the antioxidant and anti-inflammatory effect of CM on an experimental model of acute inflammation [30] and also the antioxidant effect of CM and AuNPsCM in an in vitro study, proving their efficacy on an experimental model of celiac disease [31]. We have also assessed the effects of AuNPsCM on oral dysplastic cells [32], proving the same antioxidant and anti-inflammatory effects. 

Based on our experience, we aimed to investigate the impact of CM and AuNPsCM oral administration on structural, ultrasonography, endothelial and biochemical changes, induced in the aorta and serum, by a prolonged HFD in rats.

## 2. Materials and Methods

### 2.1. Chemicals and Reagents

All the chemicals and reagents used to prepare and characterize the *Cornus mas* fruit extract and to synthesize the gold nanoparticles (acetone, HAuCl_4_, the Folin-Ciocalteu reagent, NaOH and Na_2_CO_3_) were purchased from Merck (Darmstadt, Germany). The reagents used for TEM were of analytical grade and were bought from different institutions as follows: NaH_2_PO_4_·H_2_O and Na_2_HPO_4_·12H_2_O were purchased from Reactiv (Bucharest, Romania); glutaraldehyde from Agar Scientific (Stansted, UK); OsO_4_ and EMBED 812 resin from Electron Microscopy Sciences (Hatfield, PA, USA); ethanol from VWR Chemicals (Fontenay-sur-Bois, France); uranyl acetate from Merck (Darmstadt, Germany); and lead citrate from Fluka (Buchs, Switzerland). The chemicals used for the aorta wall and serum parameters were purchased from distinct institutions: catalase and malondialdehyde colorimetric assay kit (TBA method) from Elabscience Biotechnology Inc. A (USA); Rat ET-1 (Endothelin 1) ELISA Kit, Rat NOS2/iNOS (Nitric Oxide Synthase 2, Inducible) ELISA Kit and Rat TNF-α ELISA Kit from ORION Biologics S.R.L. (Cluj-Napoca, Romania); and the reagents for C reactive protein, triglycerides, cholesterol, LDL, HDL and gamma-GT determinations from Biosystems S.A. Costa Brava (Barcelona, Spain).

Preparation of the solutions for TEM: the 2.7% glutaraldehyde solution for prefixation of aorta samples was prepared 30 min before the collection of sample, as follows: 2 mL 25% glutaraldehyde (Electron Microscopy grade) was mixed with 10 mL 0.1 M phosphate buffer (pH = 7.4) and 4.2 mL MiliQ Millipore (Burlington, MA, USA) filtered bi-distilled water (obtained with a Fistreem Cyclon water distiller, Cambridge, UK) and kept on ice (made with a Maxima ice flakes maker, Mijdrecht, The Netherlands) until use. The 0.1 M phosphate buffer used for the prefixation and washing of samples was prepared as follows: 1.56 g NaH_2_PO_4_∙2H_2_O and then 18.036 g Na_2_HPO_4_∙12H_2_O (both weighted on a Sartorius R2000D balance, Göttingen, Germany) were dissolved in 300 mL filtered bi-distilled water on a Velp Scientifica magnetic stirrer (Usmate Velate, MB Lombardy, Italy). The solution volume was corrected with filtered bi-distilled water to 495 mL, and the solution pH was adjusted to 7.4 with a Corning Pinacle 542 pH-meter (New York, NY, USA). The final volume of solution was established to 500 mL with filtered bi-distilled water and the solution was preserved at 4 °C until use. The 0.15 M phosphate buffer used for the postfixation of samples was prepared in similar conditions, but by dissolving in the filtered bi-distilled water of 2.366 g NaH_2_PO_4_∙2H_2_O and 27.139 g Na_2_HPO_4_∙12H_2_O. The 1% osmium tetroxide (OsO_4_) solution used for postfixation was prepared as follows: a glass vial containing 0.5 g of crystalline OsO_4_ was washed with detergent and cleaned several times with filtered bi-distilled water. The intact vial was introduced into a dark glass bottle containing 49.5 mL 0.15 M phosphate buffer, and the bottle was vigorously shaken until the vial broke; from time to time, the bottle was shaken for 5 min (over 24 h) until the complete dissolving of the OsO_4_ crystal. The 4 constituents of the EMBED 812 epoxy resin, kept at 4 °C, were brought to laboratory temperature; then the resin was prepared into a Berzelius glass as follows: 29.75 mL EMBed 812 were mixed with 19.6 mL dodecenyl succinic anhydride specially distilled, 14.7 mL methyl-5-norbornene-2,3-dicarboxylic anhydride and 1 mL 2,4,6 tri (dimethylaminomethyl) phenol. The solution was well homogenized prior to use.

### 2.2. Fruit Extract Preparation and Characterization

*Cornus mas* L. fruits were purchased from Central Market Cluj-Napoca in August 2021, packed in polyethylene bags and frozen till use. The vegetal material was identified by botany lecturer Irina Ielciu. A sample of the plant material was deposited in the Herbarium of Pharmaceutical Botany Department (voucher 06/2022) Faculty of Pharmacy, Iuliu Hatieganu University of Medicine and Pharmacy Cluj-Napoca, Romania. *Cornus mas* L. extract was obtained using the following procedure: 100 g mashed fruit was mixed with 300 mL acetone, the mixture was let for 1 h while stirring at room temperature, and then it was filtered and vacuum concentrated until the total acetone elimination. The concentration of total phenols from the resulting concentrated fruit extract was determined using the Folin-Ciocalteu method [33] slightly modified [34]. Thus, 3 mL Folin-Ciocalteu reagent was added to 0.5 mL *Cornus mas* L. extract (256 times diluted) and, after 5 min, 2.4 mL Na_2_CO_3_ 0.7M solution was added. The mixture was stored in a dark environment at room temperature for 2 h, and then the absorbance at 765 nm was recorded by reference to a Control sample. The result was expressed in grams of gallic acid equivalents, (GAE)/litre of extract, using a calibration curve.

### 2.3. Gold Nanoparticles Synthesis, Characterization and Tissue Determinations

A green synthesis method was applied to obtain the gold nanoparticles by reducing the gold ions from tetrachloroauric acid with antioxidant bioactive compounds present in the fruits of *Cornus mas* L. To this end, the fruit extract (25 mL) was brought to pH = 7.5 by adding NaOH 0.1 M solution, and the obtained alkaline extract was dropwise added to 100 mL boiling HAuCl_4_ 1 mM solution. The resulted mixture was stirred for 30 min at room temperature until the colour changed from faint pink to intense purple. The obtained gold colloid was centrifuged for 30 min at 14,000 rpm, and the resulted residue consisting in AuNPs was twice washed with bi-distilled water and air dried and further characterized by appropriate techniques such as UV-vis spectroscopy (using a Perkin-Elmer Lambda 25 spectrophotometer), transmission electron microscopy (using a Hitachi Automatic H-7650 microscope operated at 120 kV), Fourier transform infrared spectroscopy (FTIR, using a Brucker Vector 22 FT-IR spectrometer) and Dynamic light scattering (using Zetasizer Malvern Nano Series, UK Nano-ZS90). The UV-Vis spectra were recorded in 1 cm pathlength quartz cuvette, and absorbance was measured between 400 and 600 nm, at a resolution of 1 nm, at room temperature. The samples were prepared by 6 x dilution with distilled water. TEM samples of the gold nanoparticles were prepared by dropping the AuNPs suspension over carbon-coated copper grids, and the water was allowed to evaporate. The FTIR spectroscopy analysis was conducted on solid samples. Three mg of the dried sample (extract or nanoparticles) were mixed with 300 mg KBr (FTIR grade); the mixture was pressed into a pellet, and the spectra were recorded in the range 4000–500 cm^−1^ at a resolution of 4 cm^−1^. The zeta potential of the AuNPs was calculated using microelectrophoresis measurements collected from the DLS instrument equipped with a He–Ne laser operating at 633 nm and an avalanche photodiode detector.

The levels of gold in the serum and aorta samples were analysed by inductively coupled plasma-optical emission spectrometry (ICP-OES) using a Perkin Elmer-OPTIMA 2100 DV spectrometer. The samples were precisely weighed and dissolved in 12 mL mixture of 8:2:2 nitric acid (65%): hydrochloric acid (35%): hydrogen peroxide (35%), using MILESTONE microwave digester. The solubilised samples were diluted at 25 mL volumetric flask, and the content of gold was measured. The gold detection was made at 267.595 nm with a detection limit of 0.004 mg/L (radial mode).

### 2.4. Animals

In a preliminary experiment, performed to study the tissue distribution of gold nanoparticles functionalized with bioactive compounds from *Cornus mas* L. extract (AuNPsCM), 7 Sprague Dawley adult female rats were used. 

For the study of AuNPsCM effects in rats on a high-fat diet, 28 Sprague Dawley adult female rats were used. The animals were purchased from Cantacuzino National Medico-Military Institute for Research and Development, Bucharest, Romania. At the beginning of the experiment, the rats were 3 months old, and their body weight was 300 ± 10 g. They were kept in cages at a standard temperature of 21 ± 2 °C and a relative humidity of 55% ± 5 and were fed only standardized lipid-rich food. At the end of the experiment, the rats’ weight increased up to 600 ± 10 g. There was ad libitum access to filtered tap water but also to the same type of food that was daily administered by gavage. The housing consisted of polysulfone type III-H open-top cages (Tecniplast Institute, Bucharest, Romania). The bedding was standard aseptic wood chip (LignocelVR; J. Rettenmaier & SohneGmBH Co. KG, Rosenberg, Germany). The experiment was approved by the Ethics Committee of the Iuliu Hatieganu University of Medicine and Pharmacy (no. 158/11.03.2019) and respected Directive 86/609/EEC. 

### 2.5. High-Fat Diet (HFD)

A standardized lipid-rich diet, 20 g/100 gbw/day by gavage, was used for the entire duration of the experiment and gave an additional 45% level of energy. As per the manufacturer’s information, the ingredients of the diet were: casein, L-cysteine, corn starch, maltodextrin, cellulose, soybean oil, lard, choline, vitamin-mineral premix, calcium carbonate, salt, monocalcium phosphate, butylated hydroxytoluene (BHT) and dye. The diet had a caloric value of 4.75 kcal/g and consisted of 24.3% crude protein, 21.2% crude fat, 11.75% crude fiber. The diet was produced by Cantacuzino National Medico-Military Institute for Research and Development, Bucharest, Romania, with the identification number ROB0001.

### 2.6. Experimental Design

Prior to the experiment, the authors studied the tissue distribution of gold nanoparticles functionalized with bioactive compounds from *Cornus mas* L. extract. For this study, one group of rats was used (*n* = 7) and received for one month with an HFD, AuNPsCM by oral gavage.

**Figure 1:**
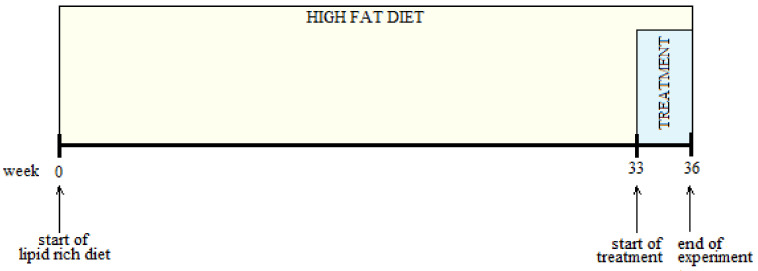
The time course of scheduled experiment to treat overweight rats after 32 weeks of HFD: at the beginning of the 33rd week with high-fat diet, the rats received by oral gavage and for another 4 weeks with HFD: normal saline 0.9% (HFD group), *Cornus mas* L. extract solution (HFD + CM group) or gold nanoparticles functionalized with bioactive compounds from *Cornus mas* L. extract (HFD + AuNPsCM group). The Control group received standard diet and, in the last month of the experiment, normal saline 0.9% by oral gavage.

For the study performed on rats with a prolonged high-fat diet, four groups (*n* = 7) were formed by randomizing 28 rats. The rats’ body weights were measured at the beginning of the experiment. After 33 weeks of high-fat diet, the treatment was introduced (Figure 1) for four weeks, according to the rats group. 

The medication was administered between 7 a.m. and 8 a.m., 0.5 mL/day, by gastric tube gavage. The Control group (with standard diet during the experiment) and HFD group received 0.9% saline solution, the group HFD + CM received *Cornus mas* L. extract (0.158 mg/mL polyphenols) and the group HFD + AuNPsCM the gold nanoparticles functionalized with bioactive compounds from *Cornus mas* L. extract (260 μg Au/kg/day). On the last day of the study, the rats’ total body weights were measured, Doppler sonography was performed, and then, blood, visceral (mesenteric) fat mass and aorta samples were collected. Blood was taken on K3EDTA tubes from the retroorbital plexus of the rats under mild anesthesia (2.5 mg/100 gbw ketamine 10% and 50 mg/100 gbw of xylazine hydroxichloride 2%), centrifuged at 6500 rpm for 10 min and kept at −80 °C until further biochemical analysis. Deep anesthesia was induced in rats, using ketamine 10% (5 mg/100 gbw) and xylazine hydroxichloride 2% (100 mg/100 gbw), to collect the visceral fat mass and descending thoracic aortas for histopathological analysis and transmission electron microscopy investigation.

### 2.7. Histopathological Examination

Tissue samples of the descending aorta were fixed in 10% neutral buffered formalin for 48 h prior to paraffin embedding and were cut into 5 μm sections using a Reichert microtome (Vienna, Austria) and stained in hematoxylin and eosin. Histopathological evaluation was performed using an Olympus BX51 microscope (Tokyo, Japan) by two independent researchers who were blinded to the experimental design of the study.

### 2.8. Transmission Electron Microscopy (TEM)

The aorta samples collected with a pair of scissors were immediately immersed in ice-cold 2.7% glutaraldehyde in 0.1 M phosphate buffer (pH = 7.4) and trimmed to ring shapes with two new razor blades (Astor, Societe BIC, Clichy Cedex, France) cutting in opposite directions. The samples were transferred into screw cape tubes (one sample/tube) with rubber O rings (Axygen Scientific, Union City, CA, USA) for 2 h prefixation at 4 °C. The samples were next washed 5 times with the same buffer (4 for 1 h each and one overnight) at 4 °C by stepwise replacing of solutions from tubes with 3 mL Pasteur pipettes (Agar Scientific, Stansted, UK) in order to remove glutaraldehyde from the cells. The samples were postfixed for 1.5 h with 1% OsO_4_ in 0.15 M phosphate buffer (pH = 7.4) at 4 °C in the same tubes. The exchange of toxic solutions (glutaraldehyde and OsO_4_) was performed under a Telstar ventilated chemical hood (Terrassa, Spain). The aorta samples were then dehydrated in an ethylic alcohol series (30–100% 30 min each) at 4 °C until 70% and the others at laboratory temperature. Next, the samples were embedded in EMBED 812 epoxy resin, after a previous infiltration with increasing ethylic alcohol solutions of resin (33%, 50% and 66% for 1 h each and 100% over-night at laboratory temperature). After the last change of pure resin, the aorta rings were transferred into 0.68 mL gelatine capsules for electron microscopy (Merck, Darmstadt, Germany), filled with fresh pure resin, and labels on filter paper (Filters Fioroni, Ingré, France) were introduced in each capsule. Polymerization occurred for 72 h at 60 °C in a Bergmann-Altmann oven (Berlin, Germany). The polymerized blocks were trimmed with single-edge carbon steel blades (Electron Microscopy Sciences, Hatfield, PA, USA) under an IOR stereomicroscope (Bucharest, Romania). The blocks were cut with glass knives (made by us with a 7801A LKB knife maker, Stockholm, Sweden) on a Bromma 8800 ULTRATOME III ultramicrotome (LKB, Stockholm, Sweden). Ultrathin sections of 60–80 nm were collected on 3 mm and 300 mesh copper grids (Agar Scientific, Stansted, UK), previously covered with a thin film of formvar (Electron Microscopy Sciences, Hatfield, PA, USA) and double contrasted: 15 min with 13% uranyl acetate in 50% ethylic alcohol and 5 min with 2.8% lead citrate. The examination of aorta sections was performed on a JEOL JEM 1011 transmission electron microscope (JEOL, Tokyo, Japan), operating at 80 kV. Images were captured using a Mega View G2 camera and an iTEM software (both from Olympus, Soft Imaging System, Münster, Germany).

### 2.9. Endothelin, iNOS and TNF-Alpha

The inflammatory factors from the aorta wall were determined using spectrometer-based ELISA readers, absorbance 450 nm, at 37 °C, and with Magellan data Analysis software, endothelin 1 and TNF-α being expressed in pg/mg protein; iNOS in ng/mg protein and MDA in nmol/mg protein.

### 2.10. Ultrasound (US) Examination

Sonography was performed using an 8–40 MHz linear array transducer on a Sonotouch Tablet System (Ultrasonix Medical Corporation, Richmond, BC, Canada), using a 20 MHz frequency for all the scans. Grey-scale, color and/or power Doppler and pulsed Doppler images were obtained for each rat included in the study. The aorta diameter was measured on transverse scans, using bidimensional US, near the level of the aortic valve. The highest anterior-posterior diameter obtained on the images was measured.

The presence of the flow in the aorta was detected using color or power Doppler US. At the pulsed Doppler examinations, the highest velocity obtained on the specific scale was measured (cm/sec).

Ventricular wall thickness was measured (in millimeters) in the transversal section, examined in grey scale, and the resulted mean size, obtained from three successive measurements on the same ventricular wall, was used in the study. The ultrasound settings were the same for all the measurements.

### 2.11. Serum Oxidative Stress Investigation

Oxidative stress parameters were investigated using the following methods: malondialdehyde (MDA) through Conti’s method [35], reduced glutahione (GSH) using Hu’s method [36] and oxidised glutathione through Vats’ method [37]. The ratio GSH/GSSG was calculated as an important indicator of oxidative stress.

### 2.12. Serum Biochemical Parameters

Several biochemical parameters (C reactive protein, triglycerides, cholesterol, HDL, LDL and gamma-GT) were examined using the BioSystems A15 analyzer with specific reagents for every investigated blood element.

### 2.13. Statistical Analysis

The obtained values were analysed with GraphPad Prism version 5.03 for Windows, GraphPad Software (San Diego, CA, USA) using one-way ANOVA followed by the Post-test Tukey. Two-way ANOVA followed by the Bonferroni post-tests was used to analyse the increase of rats’ body weight during the experiment. The significance level was set at *p* < 0.05.

## 3. Results

### 3.1. Characterization of Gold Nanoparticles Functionalized with Cornus mas L. Phytocompounds 

**Figure 2:**
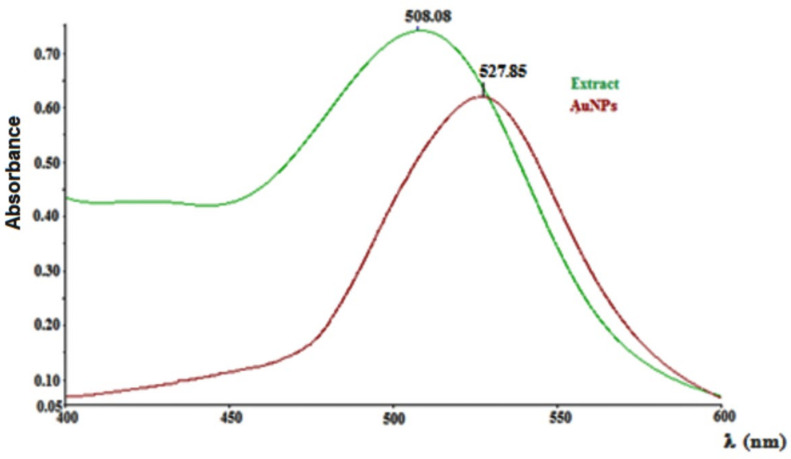
UV-Vis spectra of *Cornus mas* L. extract and gold nanoparticles.

The total content of polyphenols from *Cornus mas* L. extract, determined through Folin-Ciocalteu method, was 11.35 ± 0.48 g GAE/L extract. The UV-Vis spectrum of *Cornus mas* L. extract (Figure 2) presented a maximum at 508 nm, characteristic for the anthocyanin compounds of these fruits, while the spectrum of colloidal gold solution presented a peak at 528 nm (λ_max_), a value that is characteristic for its surface plasmon resonance [32]. 

**Figure 3:**
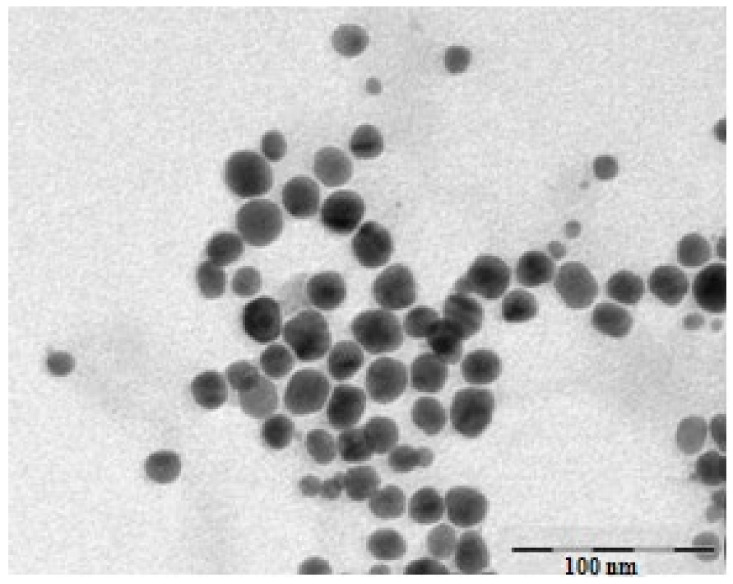
TEM image of obtained gold nanoparticles.

The TEM image of the obtained gold nanoparticles revealed their shape and morphology as well as their size. The functionalized AuNPs were spherical in shape, dispersed and presented a mean diameter of 19 nm (Figure 3).

**Figure 4:**
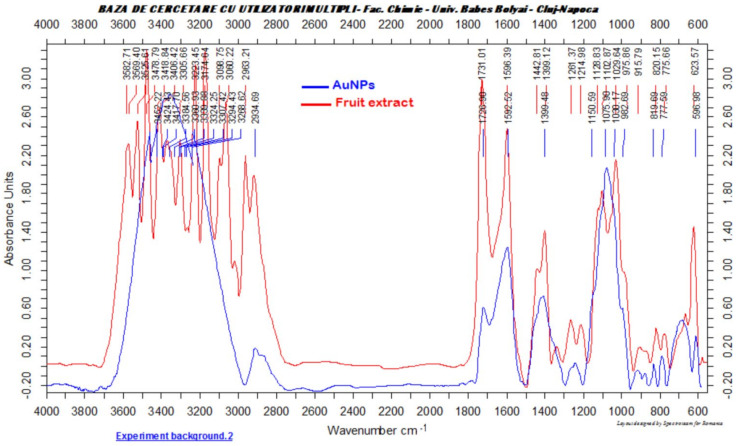
FTIR spectra of the obtained gold nanoparticles and biomolecules from the fruit extract.

The FTIR analysis was conducted aiming the confirmation of the presence of the biomolecules from the *Cornus mas* fruit extract at the surface of the obtained metallic nanoparticles, molecules which act as stabilizing agents of the AuNPs. FTIR is a useful tool that presents a clear evidence of the presence of organic moieties at the surface of the nanoparticles [38,39,40]. Figure 4 depicts the FTIR spectrum of the green synthesized gold nanoparticles compared to the spectrum of the biomolecules from the fruit extract.

Similar absorption bands were observed in the two presented spectra. A broad absorption band at 3418 cm^−1^, characteristic for the OH stretching vibration in phenolic compounds, was observed in the FTIR spectrum of the fruit extract, the same band, slightly shifted at 3424 cm^−1^, being present in the FTIR spectrum of the AuNPs. The C = O stretching vibration could be observed at 1727 cm^−1^ in the spectrum of the AuNPs, while in the fruit extract spectrum, the same band was present at 1731 cm^−1^. The aromatic moiety of the phenolic compounds can also be confirmed by the presence of the C = C vibration, which can be observed at 1592 cm^−1^ in the AuNPs FITR spectrum and at 1596 cm^−1^ in the fruit extract spectrum. The obtained results demonstrated that organic compounds, especially phenolics such as flavonoids and anthocyanins from the fruit extract, are present at the surface of the AuNPs, enhancing their biological activities (Figure 4).

The measured value of the zeta potential of the synthesized nanoparticles was −21.9 mV, proving that their surface was negatively charged and they were stable enough to be used in biological determinations.

### 3.2. Preliminary Experiment: AuNPsCM Tissue Distribution 

The AuNPsCM levels in the serum and aorta wall were searched to verify the presence of functionalized gold nanoparticles. In serum, 0.061 ± 0.008 mg/L, and in the aorta wall, 0.032 ± 0.003 mg/g were determined.

### 3.3. Experiments in Rats with a High-Fat Diet

#### 3.3.1. Weight Gain in Rats

**Figure 5:**
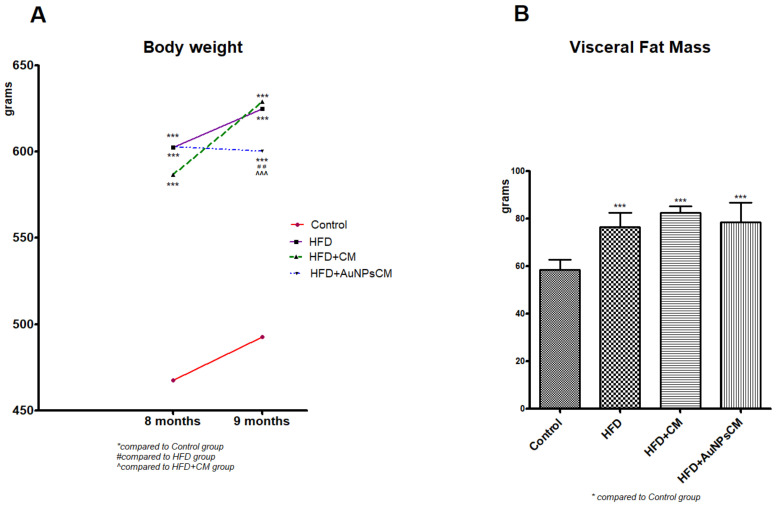
(**A**) Dynamics of rats’ body weight during the 9 months of HFD, the last month with oral administration of: 0.9% saline solution for Control and HFD groups, *Cornus mas* L. extract for HFD + CM group and gold nanoparticles functionalized with bioactive compounds from *Cornus mas* L. extract for HFD + AuNPsCM group. The results were statistically analyzed by two-way ANOVA followed by the Bonferroni post-tests, using GraphPad Prism version 5.03 software (GraphPad, San Diego, CA, USA). The parameters were expressed as means ± SD (*** *p* < 0.001 compared to Control group, ## *p* < 0.01 compared to HFD group; ^^^ *p* < 0.001 compared to HFD + CM group). (**B**) Modifications of visceral fat mass in rat groups. The results were statistically analyzed by one-way ANOVA followed by the Post-test Tukey, using GraphPad Prism version 5.03 software (GraphPad, San Diego, CA, USA). The parameters were expressed as means ± SD (*** *p* < 0.001 compared to Control group).

All the rats survived the experiment, and their weight was measured at the beginning of the experiment and after 8 and 9 months (before and after the treatment administration). Compared to the Control group, the test groups presented significant increases in body weight (*p* < 0.001). At the end of the experiment, among the test groups, considerable modifications were recorded: significant decreases of body weight in rats that received AuNPs + CM compared to the HFD group (*p* < 0.01), but also in comparison with the HFD + CM group (*p* < 0.001) (Figure 5A). The visceral fat mass increased significantly (*p* < 0.001) in all the rats with a fat lipid diet, with or without medication, with no significant modifications among the test groups (Figure 5B).

#### 3.3.2. Aorta Investigations

##### Histological Examination

**Figure 6:**
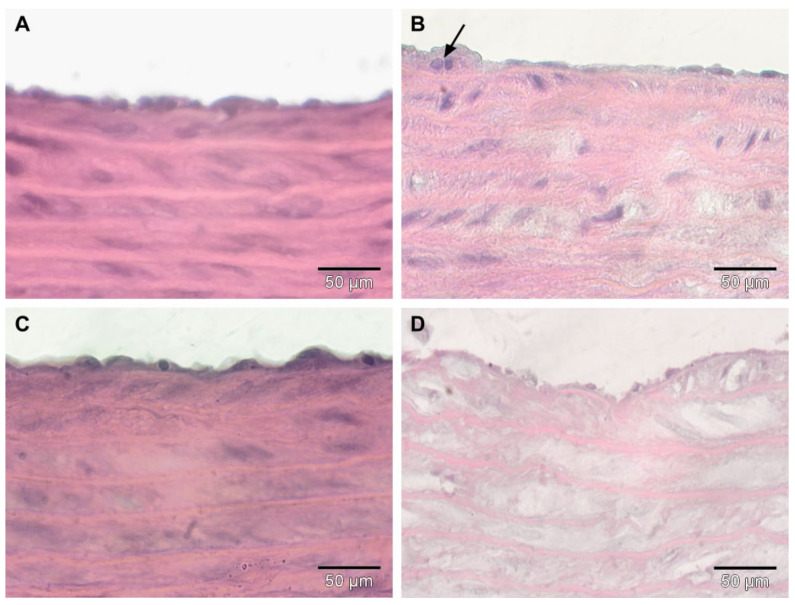
Haematoxylin-eosin staining of descending aorta of rats with 9 months of HFD, the last month with oral gavage treatment (0.9% saline solution for Control and HFD groups; *Cornus mas* L. extract for HFD + CM group; gold nanoparticles functionalized with bioactive compounds from *Cornus mas* L. extract for HFD + AuNPsCM group) showed normal morphology in Control group (**A**), macrophages (arrow) in subendothelial connective tissue layer in HFD group (**B**), thinner elastic laminae in HFD + CM group (**C**) and discontinued intima, rarefied elastic layers and several large intercellular spaces in HFD + AuNPsCM group (**D**).

In the *Control group* (Figure 6A), histological examination showed the normal morphology of the aorta with the intact endothelium and a thick media with a considerable amount of elastin fibers. In the *HFD group*, the subendothelial connective tissue layer was infiltrated with macrophages (arrow) (Figure 6B). The *HFD + CM group* (Figure 6C) showed a similar morphology to that observed in the Control group (Figure 6A), with the exception of thinner elastic laminae. In the group in which HFD was associated with functionalized gold nanoparticles (*HFD + AuNPsCM*), the intima was discontinued in many regions, with elastic layers appearing to be rarefied and with large intercellular spaces in some areas (Figure 6D).

##### TEM Investigation

**Figure 7:**
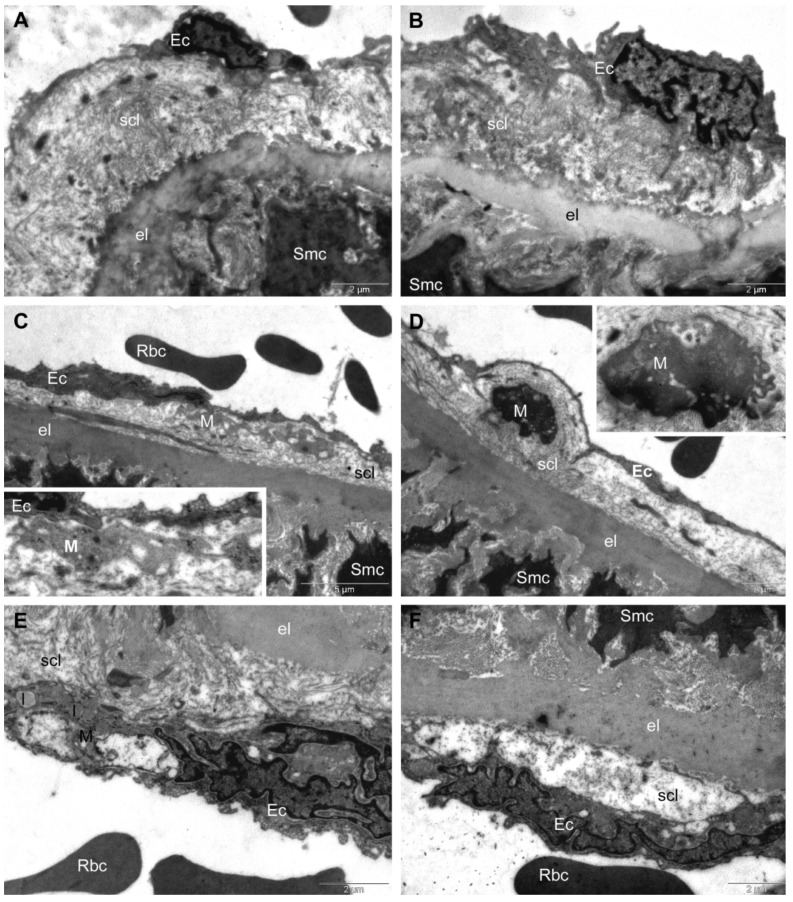
TEM investigation of descending aorta of rats with HFD for 9 months; the last month with oral gavage treatment (0.9% saline solution for Control and HFD groups) showed normal ultrastructure in Control group (**A**,**B**). In HFD group, TEM investigation showed: thinner intima, subendothelial connective layer infiltrated by macrophages with lipid inclusions (**C**,**D**), foam cells (**E**) and tendency of separation of the endothelial cells from the subendothelial connective layer (**F**). (Ec, endothelial cell; Rbc, red blood cell; scl, subendothelial connective layer; Smc, smooth muscle cell; M, macrophages; l, lipid inclusion).

In the *Control group*, the TEM investigation revealed normal ultra-structures. Intima presented endothelial cells with extensions that delineated the vascular lumen into which red blood cells could be observed, and it was separated from media by the first elastic lamina. The subendothelial connective layer contained mainly collagen fibres. Aorta media consisted of many musculo-elastic layers (with smooth muscle cells), separated by concentric elastic laminae (not shown) (Figure 7A,B).

In the *HFD group*, the aortas of rats showed a much thinner intima, reduced to less than a half of its normal thickness. The endothelial cells displayed normal ultrastructure, but in many of them, a higher number of transcytosis vesicles was noted. The subendothelial connective layer was thinned and infiltrated with macrophages containing lipid inclusions under their transformation into foam cells (Figure 7C–E). Beneath the endothelium, and sometimes in contact with endothelial cells, foam cells were often present that were filled with relatively large lipid droplets (Figure 7E). In all examined sections, the subendothelial connective layer contained foam cells with irregular shapes, with smaller or larger amorphous lipid inclusions with a heterogeneous aspect. In some regions, we noted a tendency of separation of the endothelial cells from the subendothelial connective layer, which also appeared rarefied (Figure 7F). No ultrastructural changes were recorded in media of the rats in this group; both the smooth muscle cells and the elastic laminae separating the muscle layers presented normal aspects.

**Figure 8:**
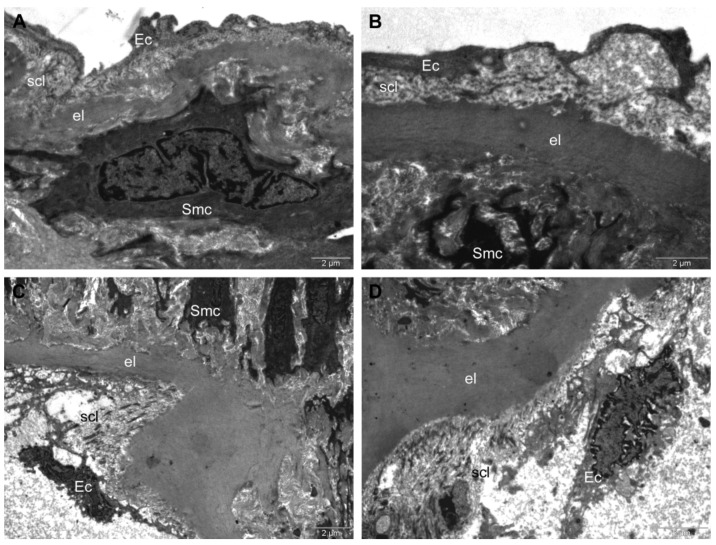
TEM investigation of descending aorta of rats with HFD for 9 months; the last month with oral gavage treatment (*Cornus mas* L. extract for HFD + CM group and gold nanoparticles functionalized with bioactive compounds from *Cornus mas* L. extract for HFD + AuNPsCM group) showed normal intima aspect, but thinned subendothelial connective layer in HFD + CM group (**A**,**B**). In HFD + AuNPsCM group, TEM investigation showed: deeply altered endothelial cells that were weakly connected to the elastic lamina; extensive denudation of inner elastic lamina; disorganized subendothelial connective layer with lipid inclusions and almost completely disrupted collagen network (**C**,**D**). (Ec, endothelial cell; el, elastic lamina; scl, subendothelial connective layer; Smc, smooth muscle cell; l, lipid inclusion.)

In the *HFD + CM group*, the intima displayed a normal aspect, but with a thinned subendothelial connective layer (Figure 8A,B).

In the *HFD + AuNPsCM group*, the disorganization of the intima structure was found: the endothelial cells were deeply altered with the denudation of the inner elastic lamina in extensive areas, along with the disorganization of the subendothelial connective layer. Despite the fact that the collagen network was still attached to the internal lamina, it was almost completely disrupted, and the endothelial cells were only weakly connected to the elastic lamina. In the disorganized subendothelial layer, the heterogeneous lipid inclusions were visible. The smooth muscle cells were not affected (Figure 8C,D).

##### Biochemical Parameters from Aorta Homogenates

Parameters to assess oxidative stress and inflammation were evaluated from the aorta homogenates.

**Figure 9:**
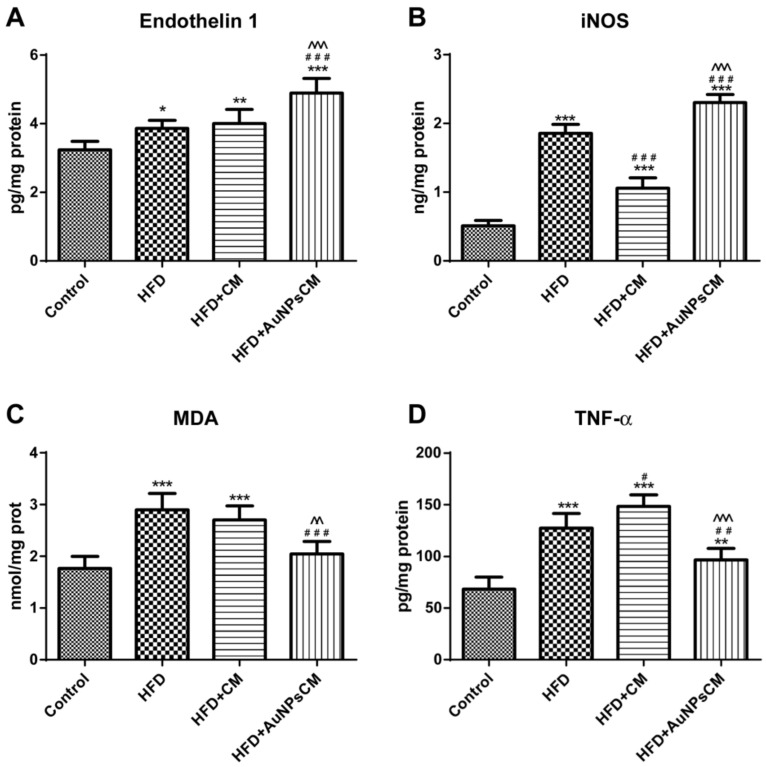
The aorta parameters after 9 months of HFD, the last month with oral administration of: 0.9% saline solution for Control and HFD groups, *Cornus mas* L. extract for HFD + CM group and gold nanoparticles functionalized with bioactive compounds from *Cornus mas* L. extract for HFD + AuNPsCM group: endothelin 1 (**A**), iNOS (**B**), MDA (**C**) and TNF-alpha (**D**) (* compared to Control group, # compared to HFD group; ^ compared to HFD + CM group). The results were statistically analyzed by one-way ANOVA followed by the Post-test Tukey, using GraphPad Prism version 5.03 software (GraphPad, SanDiego, USA). The parameters were expressed as means ±SD (* *p* < 0.05, ** *p* < 0.01, *** *p* < 0.001 compared to Control group; ## *p* < 0.01, ### *p* < 0.001 compared to HFD group; ^^ *p* < 0.01, ^^^ *p* < 0.001 compared to HFD + CM group).

Endothelin 1 (ET1) levels were increased in all the test groups, compared to the Control group: HFD (*p* < 0.05), HFD + CM (*p* < 0.01) and HFD + AuNPs + CM (*p* < 0.001). AuNPs functionalized with *Cornus mas* L. phytocompounds produced the highest increase of ET1 levels (compared to HFD and HFD + CM groups: *p* < 0.001) (Figure 9A). Nitric oxide synthase (iNOS) had significant increases in test groups (*p* < 0.001) in comparison with the Control group, but compared to the HFD group, iNOS presented significant decreases (*p* < 0.001) in the HFD + CM group and significant increases (*p* < 0.001) in the HFD + AuNPsCM group. Gold nanoparticles functionalized with bioactive compounds from *Cornus mas* L. induced a greater synthesis of iNOS than the simple *Cornus mas* L. extract administration (*p* < 0.001) (Figure 9B). In the aorta, lipid peroxidation was determined by MDA levels that were significantly increased in rats of the HFD and HFD + CM groups, compared to the Control group (*p* < 0.001). AuNPsCM administration decreased significantly the MDA levels in rats with this medication, compared to those with the HFD, without treatment (*p* < 0.001) or with *Cornus mas* L. simple extract (*p* < 0.01) (Figure 9C). Tumour Necrosis Factor alpha (TNF-α) was investigated in aorta samples and showed significant increases, compared to the Control group, in HFD (*p* < 0.001), HFD + CM (*p* < 0.001) and HFD + AuNPsCM (*p* < 0.01) groups. Compared to the rats with only a high-lipid diet, *Cornus mas* L. extract produced significant increases (*p* < 0.05) in simple oral administration but significant decreases (*p* < 0.01) when administered as a nanoparticulate formulation (Figure 9D).

##### Ultrasound Examination of the Aorta and Left Ventricle

**Figure 10:**
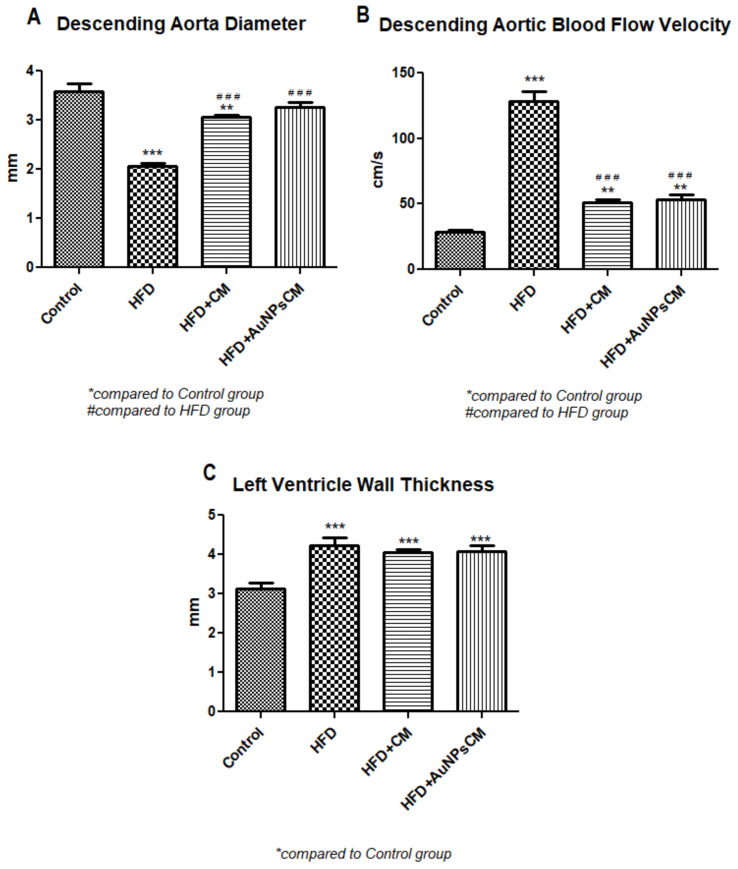
The variations of diameter (**A**) and blood flow velocity (**B**) in the descending aorta and the left ventricle thickness (**C**) modifications in rats with 9 months of HFD, the last month with oral administration of: 0.9% saline solution for Control and HFD groups, *Cornus mas* L. extract for HFD + CM group and gold nanoparticles functionalized with bioactive compounds from *Cornus mas* L. extract for HFD + AuNPsCM group (* compared to Control group, # compared to HFD group). The results were statistically analyzed by one-way ANOVA followed by the Post-test Tukey, using GraphPad Prism version 5.03 software (GraphPad, San Diego, CA, USA). The parameters were expressed as means ± SD (** *p* < 0.01, *** *p* < 0.001 compared to Control group; ### *p* < 0.001 compared to HFD group).

**Figure 11:**
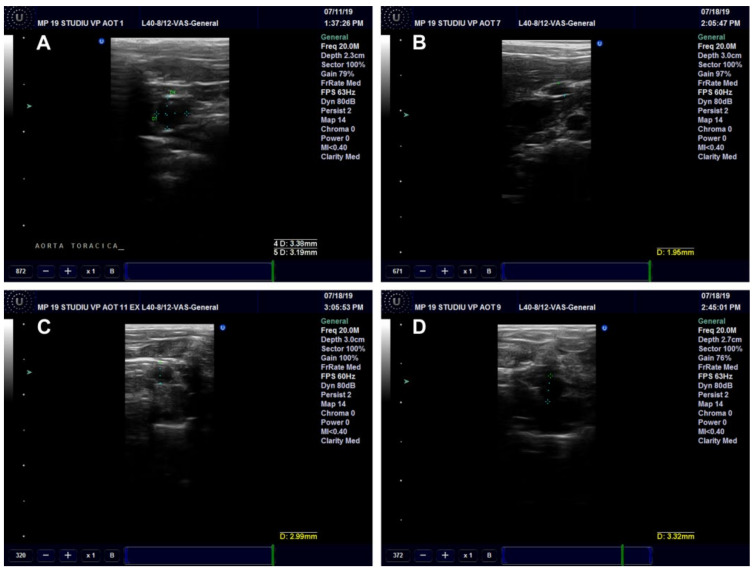
Ultrasound examination of the descending aorta diameter in rats with 9 months of HFD, the last month with oral administration of: 0.9% saline solution for Control (**A**) and HFD (**B**) groups, *Cornus mas* L. extract for HFD + CM group (**C**) and gold nanoparticles functionalized with bioactive compounds from *Cornus mas* L. extract for HFD + AuNPsCM group (**D**).

**Figure 12:**
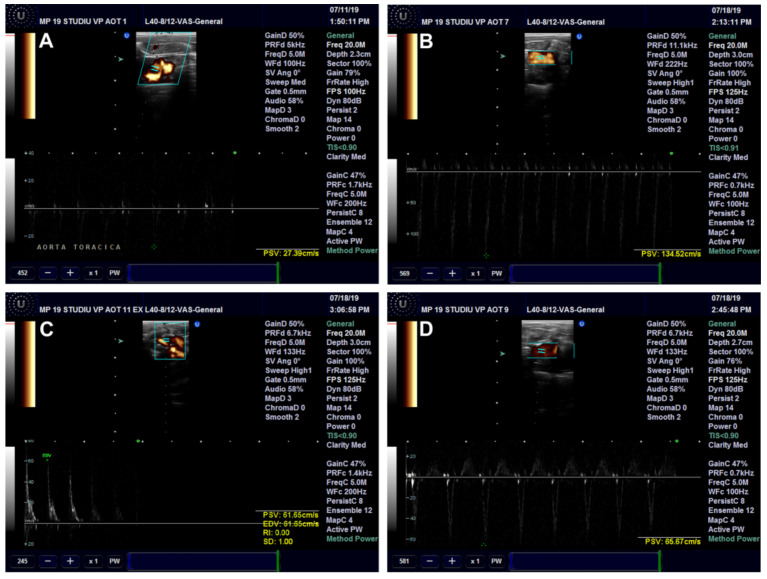
Descending aorta blood flow in rats with 9 months of HFD, the last month with oral administration of: 0.9% saline solution for Control (**A**) and HFD (**B**) groups, *Cornus mas* L. extract for HFD + CM group (**C**) and gold nanoparticles functionalized with bioactive compounds from *Cornus mas* L. extract for HFD + AuNPsCM group (**D**).

**Figure 13:**
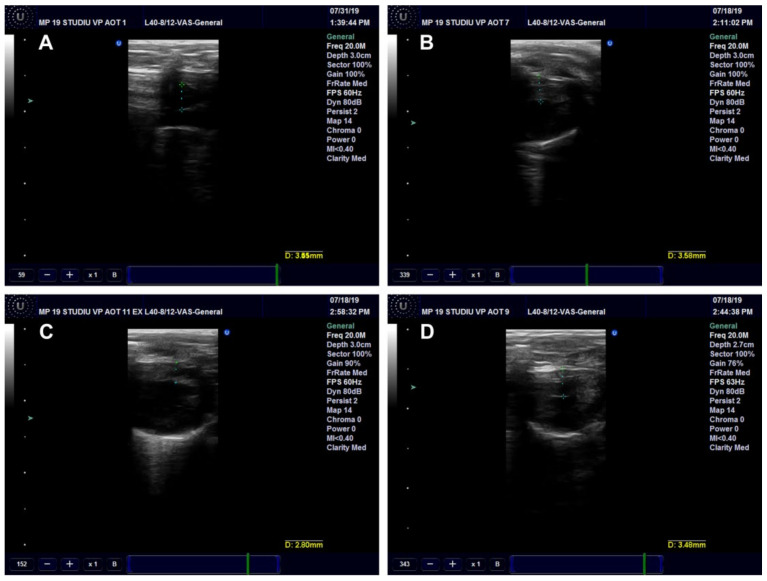
Left ventricle wall thickness in rats with 9 months of HFD, the last month with oral administration of: 0.9% saline solution for Control (**A**) and HFD (**B**) groups, *Cornus mas* L. extract for HFD + CM group (**C**) and gold nanoparticles functionalized with bioactive compounds from *Cornus mas* L. extract for HFD + AuNPsCM group (**D**).

The effects of *Cornus mas* L. administration on the aorta and left ventricle walls were investigated in the rats on a high-fat diet. The aorta diameter was significantly reduced in the HFD (*p* < 0.001) and HFD + CM (*p* < 0.01) groups as compared to the Control group. Compared to the HFD group, both types of *Cornus mas* L. administration produced significant increases (*p* < 0.001) of aorta diameter (Figure 10A and Figure 11). The blood flow rate was measured in the descending aorta, and the highest velocity was recorded in HFD group (*p* < 0.001), with significant increases being also seen in the groups that received *Cornus mas* L. extract (*p* < 0.01) compared to the Control group. The administration of natural extract and of gold nanoparticles decreased significantly the descending aortic flow (*p* < 0.001), in comparison with the HFD group (Figure 10B and Figure 12). The impact of aorta modifications on the left ventricle wall was also investigated, and the measurements showed significant increases (*p* < 0.001) of the ventricle thickness in all test groups, compared to the Control group (Figure 10C and Figure 13), but the values were slightly decreased by CM and AuNPsCM administration, even though there was no statistical significance between the groups.

#### 3.3.3. Serum Examination

##### Serum Oxidative Stress Parameters

**Figure 14:**
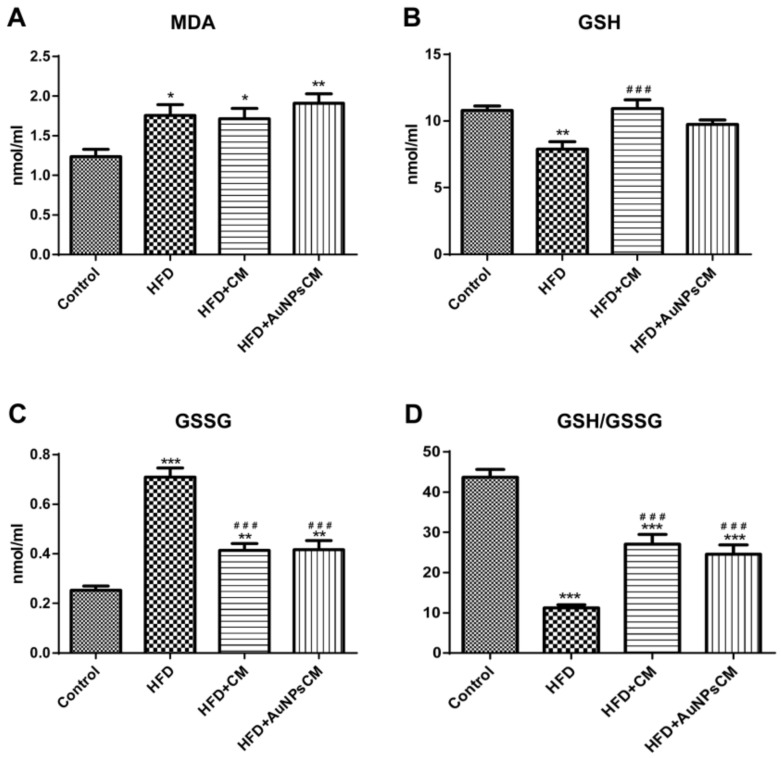
The serum oxidative stress parameters after 9 months of HFD, the last month with oral administration of: 0.9% saline solution for Control and HFD groups, *Cornus mas* L. extract for HFD + CM group and gold nanoparticles functionalized with bioactive compounds from *Cornus mas* L. extract for HFD + AuNPsCM group: malondialdhyde (MDA) (**A**), reduced glutathione (GSH) (**B**), oxidized glutathione (GSSG) (**C**) and ratio GSH/GSSG (**D**) (* compared to Control group, # compared to HFD group). The results were statistically analyzed by one-way ANOVA followed by the Post-test Tukey, using GraphPad Prism version 5.03 software (GraphPad, San Diego, CA, USA). The parameters were expressed as means ± SD (* *p* < 0.05, ** *p* < 0.01, *** *p* < 0.001 compared to Control group; ### *p* < 0.001 compared to HFD group).

Lipid peroxidation, investigated through malondialdehyde (MDA), was increased significantly in the serum of test groups rats, compared to the Control group (*p* < 0.05 for HFD and HFD + CM groups), with higher levels of MDA in the rats that received gold nanoparticles functionalized with bioactive compounds from *Cornus mas* L. extract (*p* < 0.01) (Figure 14A). A reduced glutathione (GSH) concentration was significantly decreased (*p* < 0.01) in rats with a high-fat diet in comparison with the Control group but significantly increased (*p* < 0.001) in the serum of rats that were treated with simple *Cornus mas* L. extract, compared to the HFD group (Figure 14B). Significant increased levels of oxidized glutathione (GSSG) were found in rats with a high-fat diet, without treatment (*p* < 0.001) or with *Cornus mas* L. different solutions (*p* < 0.01), compared to the Control group. The administration of this antioxidant reduced significantly (*p* < 0.001) the glutathione oxidation in rats with a high-fat diet, but the levels of GSSG remained at higher levels than those recorded in rats with the normal diet of the Control group (Figure 14C). The ratio GSH/GSSG decreased significantly in all the rats with a high-lipid diet (*p* < 0.001) compared to the Control group, but it was increased significantly (*p* < 0.001) in the treated groups, in comparison with the HFD group (Figure 14D).

##### Serum Biochemical Parameters

In serum, C-reactive protein (CRP) was significantly decreased in treated groups, compared to the Control group (*p* < 0.001) and to the HFD group (*p* < 0.01 for HFD + CM group (*p* < 0.001 for HFD + AuNPsCM group). Significant modifications have also been seen between the treated groups; the AuNPsCM administration decreased the CRP level (*p* < 0.001). The lipids levels presented different significant variations among the groups. Triglycerides increased significantly in untreated rats with a high-fat diet, compared to the Control group (*p* < 0.001), and decreased significantly in the treated groups compared to the HFD group (*p* < 0.001). The AuNPsCM administration decreased significantly the triglycerides levels in comparison with the Control group (*p* < 0.05) and with the HFD + CM group (*p* < 0.01) and increased significantly (*p* < 0.05) the cholesterol and HDL levels compared to the Control group. LDL levels, compared to the Control group, increased significantly in the test groups: HFD (*p* < 0.05) and in the treated rats (*p* < 0.001) (Table 1). 

## 4. Discussion

*Cornus mas* fruits are well known for their biological properties, having antioxidant, anti-inflammatory, neuro-protective, anti-tumour and hypoglycaemic effects, properties that are conferred by their high polyphenols content [41]. Since the *Cornus mas* L. fruit extract exerts antioxidant effects, it can also be used in nanoparticulate formulas to investigate the possible improved delivery to the target tissues. Practice proved that the bioactive *Cornus mas* L. compounds have not only a reductive capacity but also the property to stabilize the metallic nanoparticles, blocking their agglomeration [42].

The authors investigated the effects and the differences between the administration of *Cornus mas* L extract and AuNPs functionalized with natural bioactive compounds from this extract in overweight female rats after a prolonged high-fat diet that led to significant increases in the animal body weight. 

The administration of AuNPs functionalized with *Cornus mas* L. phytocompounds produced less weight gain, a result that is concordant with those observed by Chen et al. in their study performed in mice with pre-existing obesity with intraperitoneal injections of gold nanoparticles alone [43] and with those recorded by Zhang et al. in their experiment with AuNPs oral administrated in mice [44]. However, the results obtained by Chen et al. [43] and by Zhang et al. [44] emphasize the fact that weight loss was observed only under specific doses and through a particular route of administration.

The mesenteric fat has a lower lipid mobilization capacity, the peritoneal fat being the one that respond very quickly to the nutritional modifications, as Palou et al. presented in their study [45]. In our experiment, we focused on the abdominal fat response to the administered treatment, and we did not record any significant modifications of the visceral (mesenteric) fat levels among the groups with an HFD, results that when correlated with those of total body weight may prove, indirectly, the effect of administered medication on other fats compartments. At this point of our experiment we do not have enough data to explain these results. Further experiments are needed to understand better these discrepancies.

The present study was focused on the descending aorta and blood parameters. The preliminary study showed the presence of AuNPsCM in the serum and in the aorta wall, proving the efficacy of this nanoparticulate system in the transport and in the delivery of the medication to the target tissue. The obtained AuNPsCM diameter was small enough (19 nm) to enter freely into the endothelial cells through the 20 nm gap that was proven to exist between the proteoglycan chains of the endothelial glycocalyx [46,47], but also the endocytosis may be involved [48], especially the caveolin-mediated endocytosis, the predominant mechanism used by endothelial cells to uptake the nanoparticles [49]. 

Prolonged high-fat diet altered the intima of the rats’ aortas, leading to foam cells development, macrophages transformation after pinocytosis of high levels of LDL and cholesterol from serum, a mechanism described by Bahamonde et al. in their study [50] and by Kruth in his review [51], and also demonstrated by the present study. AuNPsCM administration significantly affected the aorta walls of rats with a high-fat diet, disorganizing the intima, effects that were visible in histological and TEM examinations. The foam cells, an important feature of early atherosclerosis lesions [52], were not visible in the aorta walls of rats with AuNPsCM treatment, the severe alteration of the intima layers showing only heterogenous inclusions. *Cornus mas* L. solution preserved the aorta walls of rats with an HFD, with some thinning effects on intima, protective effects that are also mentioned by Omelka et al. in their study performed on ZDF (Zucker diabetic fatty) rats [53]. 

Baratella et al. showed in their study that a high-lipid diet could increase the release of ET1 [54], effects that were also seen in our study in all rats with an HFD, but much more expressed in the treated groups, with the highest levels recorded in HFD + AuNPsCM group. Endothelin 1 expression is enhanced in atherosclerotic lesions and is involved in macrophages activation [55], both processes being seen in TEM examination, in all groups with an HFD. The vasocontrictory effects produced by ET1 especially in rats with AuNPsCM administration seemed to be counteracted by the nitric oxide, synthesized by the high levels of iNOS, effects that were visible in the sonography measurements (preserved aorta diameter and reduced velocity of blood flow). *Cornus mas* L. extract, compared to the HFD group, significantly increased the aorta diameter, decreasing the velocity of blood flow, results that are concordant with the CM effects on vascular smooth muscle presented by Hosseinpour-Jaghdani et al. in their review [56] and with the vasorelaxant properties of this natural compound presented by Bujor et al. in their study [57]. Inducible NO synthase (iNOS), an enzyme produced in atherosclerotic lesions [58], was significantly increased by AuNPsCM administration, compared to the HFD group. This result is concordant with the recorded rats’ altered aorta walls, where the inflammation may also be involved, through TNF-α in iNOS production, as Nussler et al. presented in their report [59]. The large amounts of nitric oxide produced by iNOS [60] can combine with superoxide to make peroxynitrate [61], an unstable reactive specie that can amplify the nitrosative stress [62], a mechanism that can be involved in the lesions produced in the aorta wall of these rats treated with AuNPsCM. *Cornus mas* L. extract administration decreased significantly the iNOS expression in HFD rats, a result that is concordant with those presented by Lietava et al. in their review [63]. Similar with the results of our previous study performed on healthy rats, AuNPsCM administration decreased the lipid peroxidation and the level of TNF-α, effects seen in the aorta wall, in HFD [64]. The decrease of TNF-α levels in AuNPsCM administration may be produced by the binding of this cytokine through S-Au bondings to the nanoparticles, as Tsai et al. demonstrated in their study [65]. 

The increase of left ventricle thickness in the rats that received HFD may be the consequence of administered diet, but it could also be related to the spontaneous transformation of cardiomyocytes in the Sprague Dawley rats’ hearts, as McAdams et al. presented in their study [66]. 

In serum, increased levels of MDA and reduced levels of GSH were recorded in the rats that received HFD, results that were also presented by Lasker et al. in their experiment performed on Wistar rats on a high-lipid diet [67]. Different studies that used AuNPs in vivo experiments present conflicting results, gold nanoparticles being pro-oxidant [68] or antioxidant [69] systems, but in our study, in the serum, we noticed an improvement in the antioxidant protection in HFD-treated rats. AuNPsCM administration decreased significantly the triglycerides compared to the HFD group, presenting effects on lipid metabolism. 

*Cornus mas* L. fruit extract oral administration as a single component protected better the aorta wall than the AuNPsCM solution. It increased significantly the serum antioxidant protection and decreased significantly the triglycerides level compared to the HFD group, effects that are similar with those presented by Sozanski et al. and Danielewski et al. in their studies performed in rabbits with a high-cholesterol diet [70,71]. It also decreased significantly the CRP, compared to the HFD group, decreasing the inflammation. 

In our experiment, the noticed effects of AuNPsCM administration may be the results of their small dimensions, negativity and surface chemistry, as Adewale et al. noticed in their review [72]. Previous studies presented the small AuNPs as largely distributed particles that pass rapidly through the vessel wall toward the organs where they are stored for different periods of time, altering the specific parameters [73], effects that can explain the histological and TEM modifications recorded in our experiment, as the AuNPsCM were small enough to enter the endothelial cells [74], being able to interfere with these cells metabolism. 

The present study has some limitations: we used only female rats, the experiment omitted the necessary time for endothelium recovery, and the nanoparticles’ elimination from the rats’ bodies was not investigated. Further studies are required to completely understand the interactions between the body and gold nanoparticles functionalized with bioactive compounds from *Cornus mas* L. extract, administered in a prolonged high-fat diet.

## 5. Conclusions

Gold nanoparticles functionalized with phytocompounds from *Cornus mas* L. fruit extract, administered by oral gavage in rats on a high-fat diet, produced encouraging results: reduction of the total body weight gain when used in a dose of 260 μg Au/kg/day, acting on other fats compartments than the mesenteric fat; in the aorta wall, AuNPsCM decreased lipid peroxidation and TNF-alpha levels; in serum, it decreased the triglycerides and CRP levels, with increased antioxidant protection. *Cornus mas* L. fruit extract had similar effects on serum biochemical parameters, but of less intensity.

Ultrastructural examination of the aortic wall showed fewer negative effects in *Cornus mas* L. extract administration, compared to AuNPsCM. The intima disorganization found in the aorta walls of rats treated with AuNPsCM raises concerns regarding the use of this formulation as a drug delivery system. More studies are needed to establish the safety of this administration in high-fat diet experimental models.

## Figures and Tables

**Table 1:** Mean and standard deviations of investigated serum biochemical parameters.

Serum Parameter	Control	HFD	HFD + CM	HFD + AuNPsCM
CRP	1.67 ± 0.21	1.46 ± 0.18	1.01 ± 0.25 ^c,d^	0.42 ± 0.17 ^c,e,g^
Triglycerides	136.30 ± 9.25	266.01 ± 31.25 ^c^	151.00 ± 38.56 ^e^	81.57 ± 32.82 ^a,e,f^
Cholesterol	61.86 ± 4.05	71.83 ± 9.78	80.43 ± 17.61	84.57 ± 15.03 ^a^
HDL	45.39 ± 3.60	57.52 ± 6.55	56.00 ± 10.29	62.24 ± 13.37 ^a^
LDL	7.48 ± 0.60	10.63 ± 1.85 ^a^	13.00 ± 3.16 ^c^	12.43 ± 1.61 ^c^
Gamma-GT	1.28 ± 0.48	1.71 ± 0.48	1.42 ± 0.53	1.71 ± 0.48

^a^ compared to Control group, *p* < 0.05; ^c^ compared to Control group, *p* < 0.001; ^d^ compared to HFD group, *p* < 0.01; ^e^ compared to HFD group, *p* < 0.001; ^f^ compared to HFD + CM group, *p* < 0.01; ^g^ compared to HFD + CM group, *p* < 0.001 (Units for CRP: ng/mL, for triglycerides, cholesterol, HDL, LDL and for gamma-GT: U/L).

## Data Availability

All of the data is contained within the article.
